# Research on the comprehensive evaluation index system of social practice in Chinese universities based on the CIPP model

**DOI:** 10.1371/journal.pone.0346058

**Published:** 2026-04-24

**Authors:** Yingping Nie, Qintong Lin, Wenlai Xia, Lihui Tu

**Affiliations:** 1 NingboTech University, Zhejiang, China; 2 Zhejiang University, Zhejiang, China; USTC: University of Science and Technology of China, CHINA

## Abstract

Social practice constitutes an essential aspect of talent development in higher education institutions, and the evaluation index system for social practice enables the quality of talent cultivation in these institutions to be determined. Grounded in the theoretical framework of the CIPP evaluation model, this study incorporates insights from 27 policy texts, core studies, and qualitative interview findings. It first identifies potential evaluation indicators for social practice, then gathers research samples via questionnaires and employs the Delphi method to assess the significance of these indicators. As a result, it establishes a social practice evaluation index system for higher education institutions, which includes four primary indicators, eleven secondary indicators, and thirty-seven tertiary indicators. Building upon this, the study utilizes the AHP to score the final evaluation index system and assign weights, thus completing the construction of the index system. The index system developed in this study not only concentrates on the process and scope of social practice activities, but also considers the internal and external factors that affect the implementation of social practice. Furthermore, it emphasizes the integration of formative and summative evaluations, offering valuable guidance for enhancing and improving social practice activities.

## Introduction

Social practice is an organized, planned, and purposeful activity conducted by Chinese universities that enables college students to engage with and serve society. It embodies an educational activity that enhances both school education and social education [1]. In 2023, universities and colleges across China mobilized over 11 million teachers and students to form more than 300,000 social practice teams; these then entered the “social classroom” [2]. With the extensive and in-depth development of social practice activities and the systematic advancement of educational evaluation reforms in the new era, it has become important to comprehensively and scientifically evaluate social practice in order to enhance its ability to nurture talent. This study adopts the CIPP evaluation model, which is suitable for social practice activities in Chinese universities, and conducts localized adjustments to it. It constructs a comprehensive evaluation index system for social practice that aligns with the organizational characteristics and cultural context of Chinese universities, consisting of four dimensions—background, input, process, and outcome of social practice implementation—and their corresponding observation points. The aim is to further enhance the systematicness and accuracy of evaluation through comprehensive and objective assessment of social practice, thereby better guiding the conduct of social practice activities.

Practice-oriented education is an indispensable component of higher education in many countries [3]. As early as the 19th century, scholars in Europe and America began to explore the concept of practice-oriented education. In 1798, American educators Maria Edgeworth and Richard Lovell Edgeworth [4] delved into the subject in their book “Practical Education,” emphasizing the cultivation of students’ ability to address practical problems through social engagement. Following John S. Brubacher’s pragmatic educational philosophy, which stated that “the purpose of higher education is to produce individuals useful to society,” [5] higher education shifted its focus towards research on the cultivation of practice-oriented talents. This has resulted in the development of various forms of practice-oriented education, such as experiential education in Japan [6,7], capability education in the UK [8,9], the dual-system [10,11] practice-oriented education concept in Germany, and service learning in the US [12]. These countries performed extensive research on methods that enhance college students’ social engagement and moral education, summarizing and generalizing various social practice models, including apprenticeship [13], dual-system [14], social service [15], and labor education [16] models, and establishing mechanisms that enable students’ full participation in social practice. The introduction of the concept of “educational evaluation” by American scholar W.R. Tyler [17] in the 1940s marked a significant development. Representative scholars such as Lee J. Cronbach [18], Scriven [19], Bloom [20], and Wiggins [21] have continuously enriched and advanced educational evaluation theories. They have proposed sophisticated evaluation concepts and projects, including the CIPP evaluation model [22] and the PISA student literacy evaluation [23], which have influenced and directed the trajectory of educational evaluation. However, on the whole, Western research tends to concentrate on the comprehensive functional enhancement of social practice, with formative evaluation being the primary approach. There is a scarcity of research on feedback and the evaluation of social practice [24]; in addition, the evaluation index system used for practice-oriented talent cultivation remains imperfect [25].

Since the Chinese government launched social practice activities in 1983, the forms of social practice in universities have continuously expanded, and the content has become increasingly enriched. Social practice has gradually evolved into an extension of the curriculum system and an essential component of talent cultivation in universities. Scholars have conducted extensive research and discussions on the connotation and scope of social practice in universities [26], as well as its functions, implementation [27], operation, management [28], assessment, and evaluation [29]. With the continuous advancement of higher education reform, Western educational evaluation culture has had a profound impact on social practice. Since 2012, research has started to evaluate social practice in a concentrated manner. This can be summarized into three main aspects: (1) Integrating social practice into the overall assessment of moral education in universities. For example, Gang Feng [30] regards social practice as an integral part of students’ daily education, management, and services, thus conducting assessments of practice-oriented talent cultivation hours and credits, and the implementation of teams. (2) Evaluating social practice from a specific dimension. For instance, Yuping Cui [31] and others proposed a system for evaluating the non-economic benefits of social practice, while Shujian Xiao [32] proposed a social practice evaluation index system for college students based on student satisfaction. (3) Introducing western educational evaluation models. Scholars mainly draw on evaluation theories such as the CIPP evaluation model [33], Kirkpatrick’s evaluation theory [34], multiple intelligence evaluation theory [35], and methods such as the Delphi method [36], factor analysis [37], analytic hierarchy process [38], and social comprehensive evaluation [39] to construct evaluation models and index systems for social practice in Chinese schools. However, the content currently used to evaluate social practice is relatively simple, with evaluation methods focusing primarily on outcomes. The evaluation dimensions are mostly based on a single student’s perspective. The construction of the evaluation index system is still in its infancy, making it difficult to comprehensively, multi-dimensionally, and quantitatively assess the implementation of social practice.

## Materials and methods

### Introduction of the CIPP model

The CIPP evaluation model, established by American scholar D. L. Stufflebeam in 1967, is a comprehensive educational evaluation model. It is also known as a decision-oriented model, emphasizing the improvement in educational evaluation. This model can evaluate educational reform activities, as well as the effectiveness of teaching and learning. Consequently, it is beneficial to refer to the CIPP evaluation model when evaluating various practical activities in higher education [40]. When drawing on this model, the study focuses on four dimensions of Sinicization-oriented innovation. Firstly, in the dimension of context evaluation, it emphasizes the education-oriented guiding role of educational theories with Chinese characteristics, clarifies the positioning of social practice in the fundamental task of fostering virtue through education, and particularly strengthens the assessment of the educational orientation and ideological guidance functions within the practical context. Secondly, regarding input evaluation, it enhances the assessment of Party-government coordination, multi-stakeholder participation, and linked support mechanisms, in response to the unique organizational structures of Chinese universities. Thirdly, in process evaluation, it integrates the concepts of “teaching benefits teachers and students alike” and “teacher–student interaction” from traditional Chinese education, and highlights the infiltrating role of teacher ethics inheritance in the practice process within the Chinese educational context. Fourthly, for outcome evaluation, it adds evaluation indicators aligned with China’s talent cultivation goals, such as the promotion and application of social practice achievements in other universities. In a broader sense, social practice for college students is “an educational activity that guides college students to delve into reality, society, and life in an organized, planned, and purposeful manner, in accordance with the requirements of higher education goals, to enhance their overall quality” [41]. Therefore, when evaluating the implementation of social practice in universities by drawing on this evaluation model, localized adaptation is indispensable to align with the institutional environment and cultural traditions of higher education in China.

The CIPP model, as a comprehensive evaluation framework, can be used to assess the efficacy of social practice activities undertaken by Chinese college students. Initially, the CIPP evaluation’s process-oriented and holistic approach aligns with the systematic nature of social practice. The model segments the evaluation process into four components: context evaluation, input evaluation, process evaluation and product evaluation. By integrating the principle of “morality education first” in Chinese educational evaluation into context evaluation and incorporating “resource coordination” into input evaluation, this study has enhanced the model’s alignment with the actual management practices of Chinese universities. Secondly, the CIPP evaluation’s formative and feedback-driven characteristics meet universities’ need to continuously enhance the effectiveness of practical experiences. The CIPP model emphasizes decision-making, process orientation, and improvement through evaluation. In this study, context evaluation is entrusted with the function of value guidance: it not only assesses the educational environment and students’ cognition but also focuses on the education-oriented orientation of practical activities. Input evaluation, as the foundation for ensuring the effectiveness of the assessment, places special emphasis on investment in resources such as bases that are unique to Chinese universities. Process evaluation aims to objectively assess students’ performance during the implementation of activities, while outcome evaluation involves evaluating the results, effectiveness, and impacts of the activity implementation to determine whether the objectives should be continued, revised, or refined [42]. Thirdly, the CIPP evaluation’s adaptability and practicality are well suited to the complexity inherent in evaluating social practice activities. By integrating the concept of “targeted governance” from Chinese educational management into the evaluation implementation, this study maintains international universality while reflecting Chinese characteristics across the four-dimensional evaluation. It extensively collects feedback and incorporates specific evaluation indicators into the overall evaluation framework, thereby facilitating all stakeholders in social practice to continuously improve and refine the implementation plans of social practice activities.

Based on the analysis provided, this study’s comprehensive evaluation of the implementation of social practice in Chinese universities encompasses four key aspects. Firstly, it involves a systematic examination of the social and campus environments in which universities conduct their social practice, with special attention paid to the new requirements for practice-based education put forward in the new era of socialism with Chinese characteristics. Secondly, it includes a thorough assessment of the faculty, funding and other supportive conditions that universities must invest in social practice, with a focus on assessing the multi-stakeholder collaborative support featuring Chinese characteristics. Thirdly, it examines the overall design of practical activities by teachers and students as well as students’ performance, innovatively incorporating the implementation effect of the “curriculum-based ideological and political education” concept into the assessment scope. Lastly, it involves an evaluation of the extent to which the goals of social practice are achieved and their impact, and constructs a multi-dimensional evaluation criterion with Chinese characteristics covering literacy improvement and practice promotion, so as to grasp the overall effect of social practice activities.

### Technology route and database

**Fig 1:**
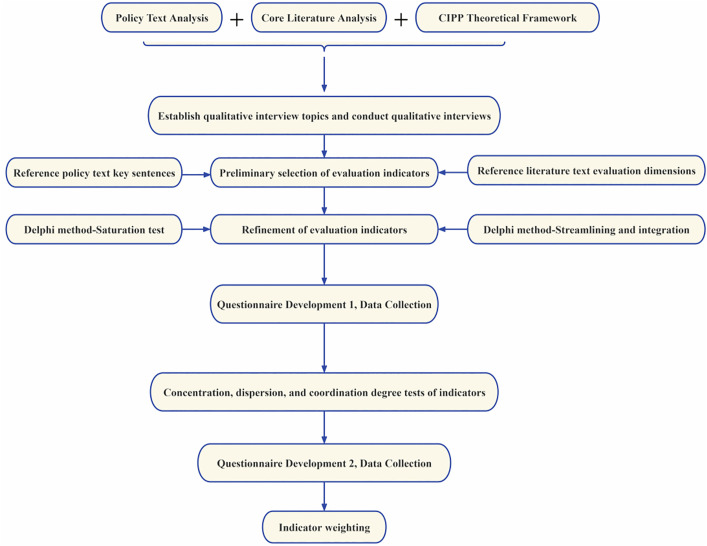
Technical Route for Indicator Selection and Validation.

To systematically construct an evaluation index system for university social practice, this study adopts a multi-stage mixed-methods research design, strictly follows the scientific process of index selection, verification, and weighting, and establishes four steps for index selection and verification (Fig 1).

First, data collection and acquisition were conducted. Six policy documents related to social practice education in Chinese universities were gathered from the websites of the Ministry of Education of the People’s Republic of China and the Central Committee of the Communist Youth League (see Table 1 in S1 Table). Additionally, twenty-one core articles on the effectiveness of social practice activities, the achievements of social practice education, and the evaluation of social practice effects in Chinese universities were selected from the CNKI database (see Table 2 in S1 Table), laying the theoretical foundation and policy basis for this study.

**Table 1:** Consistency of Expert Opinions (Secondary Indicators).

Indicator	Mean Value	Standard Deviation	Variance	Coefficient of Variation
**Social Environment (A1)**	79.231	14.641	214.359	0.185
**School Environment (A2)**	86.154	15.367	236.141	0.178
**Student Cognition (A3)**	80.615	15.851	251.256	0.197
**Faculty Support (B1)**	77.615	19.393	376.090	0.25
**Organizational Support (B2)**	80.462	15.581	242.769	0.194
**Resource Support (B3)**	81.846	15.858	251.474	0.194
**Practice Design (C1)**	77.538	20.549	422.269	0.265
**Practice Process (C2)**	81.615	15.224	231.756	0.187
**Practice Presentation (C3)**	79.615	16.601	275.590	0.209
**Direct Effects (D1)**	78.154	17.497	306.141	0.224
**Practice Impact (D2)**	78.385	17.905	320.590	0.228

**Table 2:** Consistency of Expert Opinions (Tertiary Indicators).

Indicator	Mean Value	Standard Deviation	Variance	Coefficient of Variation
**National Policy (A1-1)**	80.923	18.301	334.910	0.226
**Public Opinion Environment (A1-2)**	82.230	19.635	385.526	0.239
**Leadership Emphasis (A2-1)**	83.077	17.863	319.077	0.215
**Disciplinary Support (A2-2)**	78.769	21.549	464.359	0.274
**Campus Culture (A2-3)**	83.077	12.366	152.910	0.149
**Information Release (A2-4)**	82.462	18.528	343.269	0.225
**Regulations and Policies (A2-5)**	83.308	21.642	468.397	0.260
**Basic Cognition (A3-1)**	77.692	17.342	300.731	0.223
**Level of Understanding (A3-2)**	77.692	18.625	346.897	0.240
**Motivation for Participation (A3-3)**	76.308	21.449	460.064	0.281
**Instructor Guidance (B1-1)**	78.308	17.289	298.897	0.221
**Participation and Commitment (B1-2)**	78.615	17.741	314.756	0.226
**Specialized Institutions (B2-1)**	76.923	20.798	432.577	0.270
**Funding Allocation (B2-2)**	77.923	16.444	270.410	0.211
**Organizational Methods (B2-3)**	76.692	19.128	365.897	0.249
**Safety Assurance (B2-4)**	82.077	18.808	353.744	0.229
**Practice Bases (B3-1)**	78.385	13.080	171.090	0.167
**Training and Guidance (B3-2)**	80.077	16.363	267.744	0.204
**Activity Branding (B3-3)**	79.692	20.500	420.231	0.257
**Information Technology (B3-4)**	80.231	17.950	322.192	0.224
**Practice Theme (C1-1)**	81.077	14.192	201.410	0.175
**Practice Plan (C1-2)**	79.231	17.688	312.859	0.223
**Practice Team (C1-3)**	82.077	17.509	306.577	0.213
**Practice Implementation (C2-1)**	79.615	18.132	328.756	0.228
**Duration of Practice (C2-2)**	75.846	17.597	309.641	0.232
**Practice Performance (C2-3)**	78.385	17.993	323.756	0.230
**Team Collaboration (C2-4)**	78.692	19.076	363.897	0.242
**Practice Record (C2-5)**	75.846	21.721	471.808	0.286
**Institutional Coordination (C2-6)**	78.231	19.732	389.359	0.252
**Practice Report (C3-1)**	80.000	17.616	310.333	0.220
**Summary and Exchange (C3-2)**	79.692	17.867	319.231	0.224
**Practice Assessment (C3-3)**	82.154	16.067	258.141	0.196
**Student Evaluation (D1-1)**	78.538	15.597	243.269	0.199
**Quality Improvement (D1-2)**	83.308	8.210	67.397	0.099
**Publicity and Reporting (D2-1)**	82.385	16.566	274.423	0.201
**Awards and Recognition (D2-2)**	79.308	17.202	295.897	0.217
**Outcome Dissemination (D2-3)**	80.154	15.588	242.974	0.194

Second, qualitative interviews and preliminary index selection were conducted. Guided by the CIPP theoretical framework, one-on-one in-depth interviews were carried out with 19 participants, including university leaders, staff from the Communist Youth League Committee, social practice directors of secondary colleges, supervisors, administrative staff, and students. Using NVivo software, the study coded policy documents, core literature, and interview texts in strict accordance with the three-level coding procedure of grounded theory: first, open coding was performed to generate initial concepts; second, axial coding was adopted to establish connections between concepts; finally, selective coding was used to extract core categories. A total of 37 preliminary evaluation indicators were refined, and a theoretical saturation test was conducted to ensure the completeness of the index system.

Third, Delphi expert consultation and index refinement were conducted. This study adopted the classical Delphi method, and consensus was reached after two rounds of expert consultation. The expert panel consisted of 15 professionals with long-term experience in the management and research of university social practice, including 3 middle-level university administrators in charge of practical teaching, 5 directors of university Communist Youth League Committees with more than 10 years of work experience, 4 provincial-level outstanding instructors of social practice, and 3 scholars who have published important research results in this field. In the first round of consultation, experts’ suggestions on the modification of each element, its setting, and structure in the evaluation index system were collected. The 37 evaluation indicators were classified into 11 aspects, the importance of the evaluation indicators was tested, and the index system was revised and improved. In the second round, experts were invited to evaluate the importance of the 37 indicators across 11 categories using a 5-point Likert scale (1 = extremely unimportant to 5 = extremely important), with an open comment section set up to collect revision suggestions, achieving a 100% response rate. By calculating the mean value, standard deviation, variance, and coefficient of variation of each indicator, it was found that the mean value of all indicators exceeded 3.75 (note: the original “75” is corrected to 3.75 to match the 5-point scale logic), and the coefficient of variation was less than 0.3, indicating a high degree of consensus among the expert panel on all evaluation indicators.

Fourth, indicator weighting via the Analytic Hierarchy Process (AHP) was performed. Based on the final indicators determined by the Delphi method, this study constructed a hierarchical structure model comprising 4 first-level indicators, 11 second-level indicators, and 37 third-level indicators. The same expert panel was invited to conduct pairwise comparisons of indicators at the same level, and judgment matrices were established with reference to Saaty’s scale. Valid comparative data were collected through questionnaires, the weight of each indicator was calculated using the root mean square method, and a strict consistency test was implemented. The Consistency Ratio (CR) of all the judgment matrices was less than 0.1 (see 2.5 Indicator Weighting for details), resulting in the final construction of a complete weight system table.

### Indicator selection

Based on the results of policy text analysis and qualitative interviews, and following two rounds of Delphi expert consultation, this study finally constructed a comprehensive evaluation framework under the CIPP model, consisting of 4 first-level indicators, 11 second-level indicators, and 37 third-level indicators.

**Fig 2:**
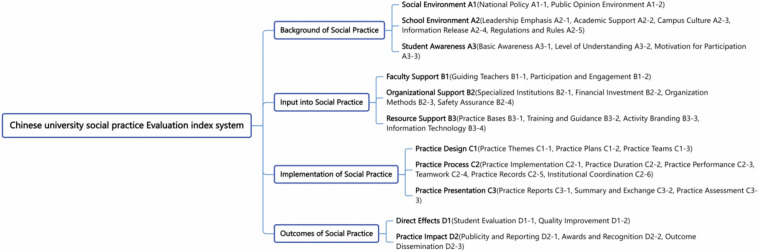
The Comprehensive Evaluation Index System for Social Practice in Chinese Universities.

The context evaluation dimension covers the social environment, school environment, and students’ cognition of social practice carried out by Chinese universities, including 3 second-level indicators (policy and public opinion environment, disciplinary support, campus culture, and students’ participation motivation) and 10 third-level indicators. The input evaluation dimension focuses on 3 second-level indicators (faculty, organization, and resource support), encompassing 10 third-level indicators such as faculty, funds, and various resources required and invested in conducting social practice. The process evaluation dimension emphasizes the implementation of practical activities, including 3 second-level indicators (theme design, program execution, and students’ performance of university social practice) and 12 third-level indicators. The outcome evaluation dimension includes the direct effects and practical impacts of social practice, consisting of 5 third-level indicators such as student evaluation, literacy improvement, and achievement promotion. The framework of the social practice evaluation index system for Chinese universities is shown in Fig 2.

### Indicator testing

Firstly, guided by the CIPP theoretical framework and based on the 37 preliminary indicators depicted in Fig 2, the study further clarified the descriptive expressions corresponding to each indicator and used these as the foundation for constructing a questionnaire (scale) regarding observation indicators. Each observation indicator was presented in the form of a declarative sentence, with five interval dimensions established: “Very Unimportant” “Unimportant” “Moderately Important” “Important” and “Very Important.” These dimensions were assigned values of “0-20” “20-40” “40-60” “60-80” and “80-100”, respectively, forming a degree scale. Secondly, employing this scale format, the study obtained opinions on the evaluation indicators through a Delphi expert survey questionnaire. Considering the reliability of the evaluation index system and the consistency of opinions, the study selected 15 individuals responsible for the implementation of social practice at Chinese universities to form a research consultation expert group. The importance of the evaluation indicators was judged according to three aspects: concentration, dispersion, and coordination. The greater the concentration, the smaller the dispersion, and the higher the coordination (the smaller the coefficient of variation), the more essential the indicator is deemed to be [43]. This approach aids in further clarifying whether to retain, delete, or modify the evaluation indicators.

#### Concentration.

Concentration ($Z_{i}$) refers to the degree of coordination among experts regarding the evaluation indicator (i), which can be expressed using Formula (1):


$$Z_{i}=\frac{\text{1}}{N}\sum_{j}^{n}k_{ij}c_{ij}$$ (1)

Here, (N) represents the total number of experts consulted; ($k_{ij}$) represents the value of the importance level (j) assigned to the evaluation indicator i(j=5,4,3,2,1); and ($c_{ij}$) represents the number of experts who have assigned the importance level (j) to the evaluation indicator (i).

#### Dispersion.

Dispersion ($D_{i}$) indicates the extent to which the experts’ opinions on the evaluation indicator (i) diverge; this can be expressed using Formula (2):


$$D_{i}=\sqrt{\frac{\text{1}}{N}\sum_{j}^{n}c_{ij}(k_{ij}-Z_{i}{)}^{\text{2}}}$$ (2)

#### Coordination degree.

Coordination Degree ($V_{i}$) indicates the extent to which the experts agree regarding the overall evaluation of the importance of the evaluation indicator (i), which can be expressed using Formula (3):


$$V_{i}=\frac{D_{i}}{Z_{i}}$$ (3)

The larger the value of ($Z_{i}$), the more important the evaluation indicator is considered to be. The smaller the value of ($D_{i}$), the more concentrated the opinions of the experts are; the converse is also true. When the results of the two cannot be consistent, it is necessary to look at the value of the coefficient of variation ($V_{i}$). The larger the value of ($Z_{i}$) and the smaller the value of($D_{i}$), the smaller the value of ($V_{i}$), indicating that the evaluation indicator is more important [44]. It should be specifically noted that when the coefficient of variation ($V_{i}$) is less than or equal to 0.3 ($V_{i}$≤0.3), there is a high degree of coordination among the expert group. When 0.3 is less than or equal to ($V_{i}$) and ($V_{i}$) is less than or equal to 0.5 (0.3≤$V_{i}$≤0.5), it indicates that the degree of coordination of opinions among the expert group is within an acceptable range. When ($V_{i}$) is greater than or equal to 0.5 ($V_{i}$≥0.5), there is a lack of agreement among the expert group, and corrections and explanations are needed [45].

The calculation of the data shows that for the 11 secondary indicators and the 37 tertiary indicators, the coefficient of variation for all items is less than 0.3. This indicates that the expert group has a high degree of coordination and agreement regarding the revised evaluation indicators, and that these indicators are of high importance. Therefore, there is no need for further the addition, deletion, or modification of the indicators. Detailed results are shown in Tables 1 and 2.

### Indicator weighting

To determine the weights of the index system, this study utilizes the AHP proposed by T.L. Satty to construct a judgment matrix for quantitative data analysis. The main steps of the method are as follows: First, a hierarchical structure model based on the aforementioned indicators is established, and pairwise comparisons are performed using a 1–5 scale to construct a pairwise comparison matrix and a judgment matrix (Table 3). Second, the AHP is employed to calculate the weight coefficients. Third, a consistency test is conducted to verify the scientific validity and rational applicability of the data obtained.

**Table 3:** 1–5 Rating Scale and Explanation.

Value (Score)	Explanation of Score
**5**	The horizontal element(i)is significantly more important than the vertical element(j).
**3**	The horizontal element(i) is slightly more important than the vertical element(j).
**1**	The horizontal element(i) is equally important as the vertical element(j).
**1/3**	The horizontal element(i) is slightly less important than the vertical element(j).
**1/5**	The horizontal element(i) is significantly less important than the vertical element(j).
**Reciprocal**	The importance of the vertical element(j) compared to the horizontal element(i).

#### Construction of the judgment matrix.

The scoring scale used in the expert consultation is a 1–5 scale, where each score corresponds to a different level of importance, as shown in Table 3:

Based on the comprehensive expert ratings, judgment matrices A, B, C, D can be formed. Taking A as an example, the final presentation is composed of score ($a_{ij}$) and satisfies the following conditions:$a_{ij}>0$; $a_{ii}=1$; $a_{ji}={1/a}_{ij}$ (*i*, *j* = 1, 2······, *n*).


$$A=\left[\begin{array}{cccc} @cccc@a_{11} & a_{12} & \cdots & a_{1j}\\ a_{21} & a_{22} & \cdots & a_{2j}\\ \cdots & \cdots & \cdots & \cdots \\ a_{i1} & a_{i2} & \cdots & a_{ij} \end{array}\right]$$ (4)

#### Calculation of weight vector.

The product of each row of the judgment matrix and its nth root are calculated to obtain the normalized eigenvector ($\omega_{i}$) and the corresponding maximum eigenvalue ($\lambda_{max}$) of the evaluation indicators. The specific calculation methods are as follows:

The matrix is normalized first by columns and then by rows. The formulas are as follows:


$$\omega_{i}=\sqrt[{n}]{\overset{n}{\underset{j}{\prod }}a_{ij}}/\sum_{i=1}^{n}\sqrt[{n}]{\overset{n}{\underset{j}{\prod }}a_{ij}}$$ (5)

Then, the maximum eigenvalue is calculated. Formulas (6) and (7) are as follows:


$$\lambda_{max}=\sum_{i=1}^{n}\frac{(A*\omega {)}_{i}}{n\omega_{i}}$$ (6)

$$(A*\omega {)}_{i}=a_{i1}\omega_{\text{1}}+a_{i2}\omega_{\text{2}}+\cdots +a_{in}\omega_{n}$$ (7)

#### Consistency check.

To ensure the scientific validity of the above steps and the rationality of the judgmental thinking, a consistency check is required. The specific procedure is as follows:

① The consistency is calculated based on the Consistency Index (CI), and the formula (8) is as follows:


$$CI=\frac{\left(\lambda_{max}-n\right)}{n-1}$$ (8)

② The value of the Random Consistency Index (RI) is determined based on the table of average random consistency indices. The specific values are shown in Table 4.

**Table 4:** Average Random Consistency Index (RI) Values.

N	1	2	3	4	5	6	7
**RI**	0	0	0.52	0.89	1.12	1.26	1.36

③ The consistency ratio is calculated using the consistency ratio formula, which is given by Equation (9):


$$CR=\frac{CI}{RI}$$ (9)

Generally, if the value of the Consistency Ratio (CR) is less than 0.1, the constructed judgment matrix has a high degree of consistency; if the value of CR is greater than 0.1, there is a significant deviation from the consistency level.

Taking experts’ weight assignments to the second-level indicators under the social practice context (A) as an example, based on each expert’s scores for the evaluation indicators, the average scores of the three observation indicators—social environment, school environment, and students’ cognition—derived from the degree of expert opinion consistency (for second-level indicators) in Table 1 are 79.231, 86.154, and 80.615 respectively.

The importance degree among various indicators is quantified using the formulaThe importance degree among various indicators is quantified using the formula aij = Si/Sj, and the calculation results are as follows:

A12=79.231/86.154=0.920  A13=79.231/80.615=0.983

A21=86.154/79.231=1.087  A23=86.154/80.615=1.069

A31=80.615/79.231=1.017  A32=80.615/86.154=0.936

Construct Judgment Matrix Table 5:

**Table 5:** Judgment matrix of secondary indicators under the social practice background A by experts.

	Social Environment A1	School Environment A2	Student Awareness A3
**Social Environment (A1)**	1	0.920	0.983
**School Environment (A2)**	1.087	1	1.069
**Student Awareness (A3)**	1.017	0.936	1

This method can maximize the retention of continuous intensity information from experts’ evaluations of indicator importance, while maintaining consistency with the 1–5 point scaling method adopted in this study in terms of data sources and logical structure. Subsequently, a consistency test is conducted on the judgment matrix, with the specific calculation process as follows:

1) According to Formula (5), the geometric mean of each row in the matrix is first calculated as (0.967,1.051,0.9837)^T^.
2) Then, the geometric mean of each row is normalized (i.e., each term is divided by the total sum) to obtain the weight vector W_i_= (0.3221,0.3502,0.3277)^T^.
3) The weight assignments of experts to the evaluation indicators are derived through the above two rounds of calculations, and the (A*W)_i_ values of the judgment matrix are calculated as (0.9664,1.0506,0.9831)^T^ according to Formula (7).
4) Substituting the above-calculated values into Formula (6), the maximum eigenvalue r_max_ is obtained as 3.0001.
5) Finally, a consistency test is performed on the obtained data to ensure the reliability of the indicator weight values. The Consistency Index (CI) is calculated as 0.00005 according to Formula (8).
6) Based on the Random Index (RI) values for multi-level judgment matrices, when n = 3, RI = 0.52. Substituting this into Formula (9), the Consistency Ratio (CR) is calculated as 0.0001 (Table 6), which is less than 0.1. This indicates that the experts’ weight distribution for the indicators is reasonable and consistent.

**Table 6:** Consistency Check of Secondary Indicators under Social Practice Background by Experts.

CI Value	RI Value	CR Value	Consistency Check Result
0.0001	0.52	0.00005	pass

After calculation, the results of all matrices from the above questionnaires meet the consistency check requirements. Therefore, it can be concluded that the single sorting of the constructed judgment matrices is consistent overall. Based on the overall sorting of various indicators, the final weight assignment results can be derived. Based on the same method described above, this study calculated the normalized weights for all indicators (see Table 7 for detailed results).

**Table 7:** Weight Assignment of the Comprehensive Evaluation Index System for Social Practice in Chinese Universities.

Weights of Primary Indicators	Weights of Secondary Indicators	Weights of Tertiary Indicators
Evaluation of Social Practice Background (16.06%)	Social Environment (3.67%)	National Policy (2.13%)
		Public Opinion Environment (1.54%)
	School Environment (4.21%)	Leadership Emphasis (0.84%)
		Disciplinary Support (0.99%)
		Campus Culture (0.68%)
		Information Release (0.58%)
		Regulations and Rules (1.12%)
	Student Awareness (8.18%)	Basic Awareness (1.97%)
		Level of Understanding (2.6%)
		Motivation for Participation (3.61%)
Evaluation of Social Practice Input (28.74%)	Faculty Support (5.86%)	Guiding Teachers (2.65%)
		Participation and Engagement (3.21%)
	Organizational Support (11.24%)	Specialized Institutions (1.85%)
		Financial Investment (3.26%)
		Organization Methods (2.9%)
		Safety Assurance (3.23%)
	Resource Support (11.64%)	Practice Bases (3.29%)
		Training and Guidance (2.87%)
		Activity Branding (2.76%)
		Information Technology (2.72%)
Evaluation of Social Practice Implementation (23.38%)	Practice Design (6.62%)	Practice Themes (1.91%)
		Practice Plans (2.01%)
		Practice Teams (2.7%)
	Practice Process (10.17%)	Practice Implementation (1.92%)
		Practice Duration (1.47%)
		Practice Performance (1.71%)
		Teamwork (1.96%)
		Practice Records (1.37%)
		University-Local Linkage (1.74%)
	Practice Presentation (6.59%)	Practice Reports (1.95%)
		Summary and Exchange (2.78%)
		Practice Assessment (1.86%)
Evaluation of Social Practice Outcomes (31.82%)	Direct Effects (15.59%)	Student Evaluation (5.51%)
		Quality Improvement (10.08%)
	Practice Impact (16.23%)	Publicity and Reporting (5.54%)
		Awards and Recognition (5.08%)
		Outcome Dissemination (5.61%)

## Results

### Determining indicator weights

Based on Equations (4)–(9), the hierarchical weight values of the evaluation index system for each expert can be calculated. The weights of each indicator in the comprehensive evaluation index system of social practice in Chinese universities are ultimately represented by the average values of all experts, as shown below in Table 7.

## Discussion

**Fig 3:**
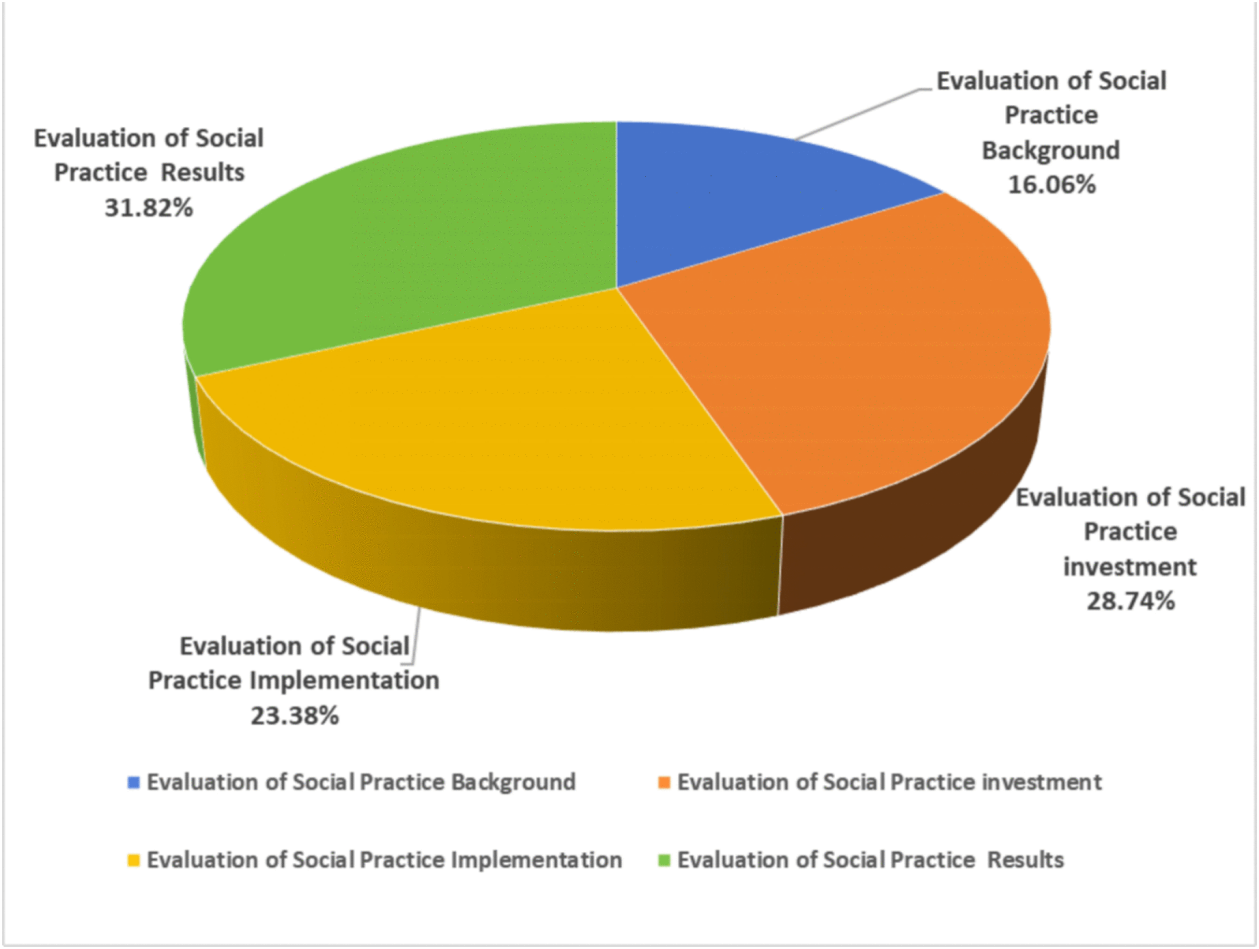
Weight of Primary Indicators in the Comprehensive Evaluation of Social Practice in Chinese Universities.

**Fig 4:**
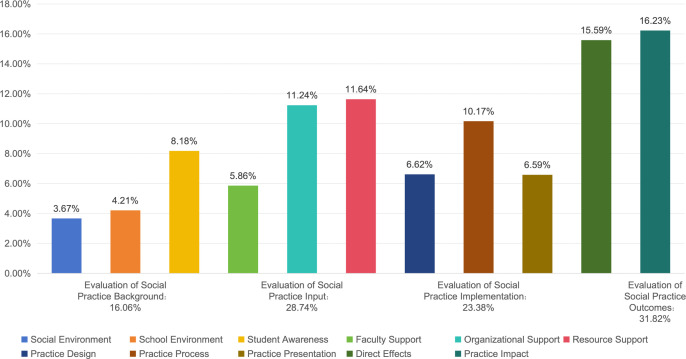
Weight of Secondary Indicators in the Comprehensive Evaluation of Social Practice in Chinese Universities.

The size of the weight values corresponding to the evaluation indicators can intuitively reflect the importance of these indicators in the overall implementation of social practice (Figs 3 and 4). It also clarifies the priorities, difficulties, and key points associated with the performance of social practice activities in Chinese universities. In the evaluation system, the four primary evaluation indicators are ranked by their weight assignments as follows: Social Practice Outcomes (31.82%)> Social Practice Input (28.74%)> Social Practice Process (23.38%)> Social Practice Background (16.06%).

### Discussion on the indicators and weights of social practice background

**Fig 5:**
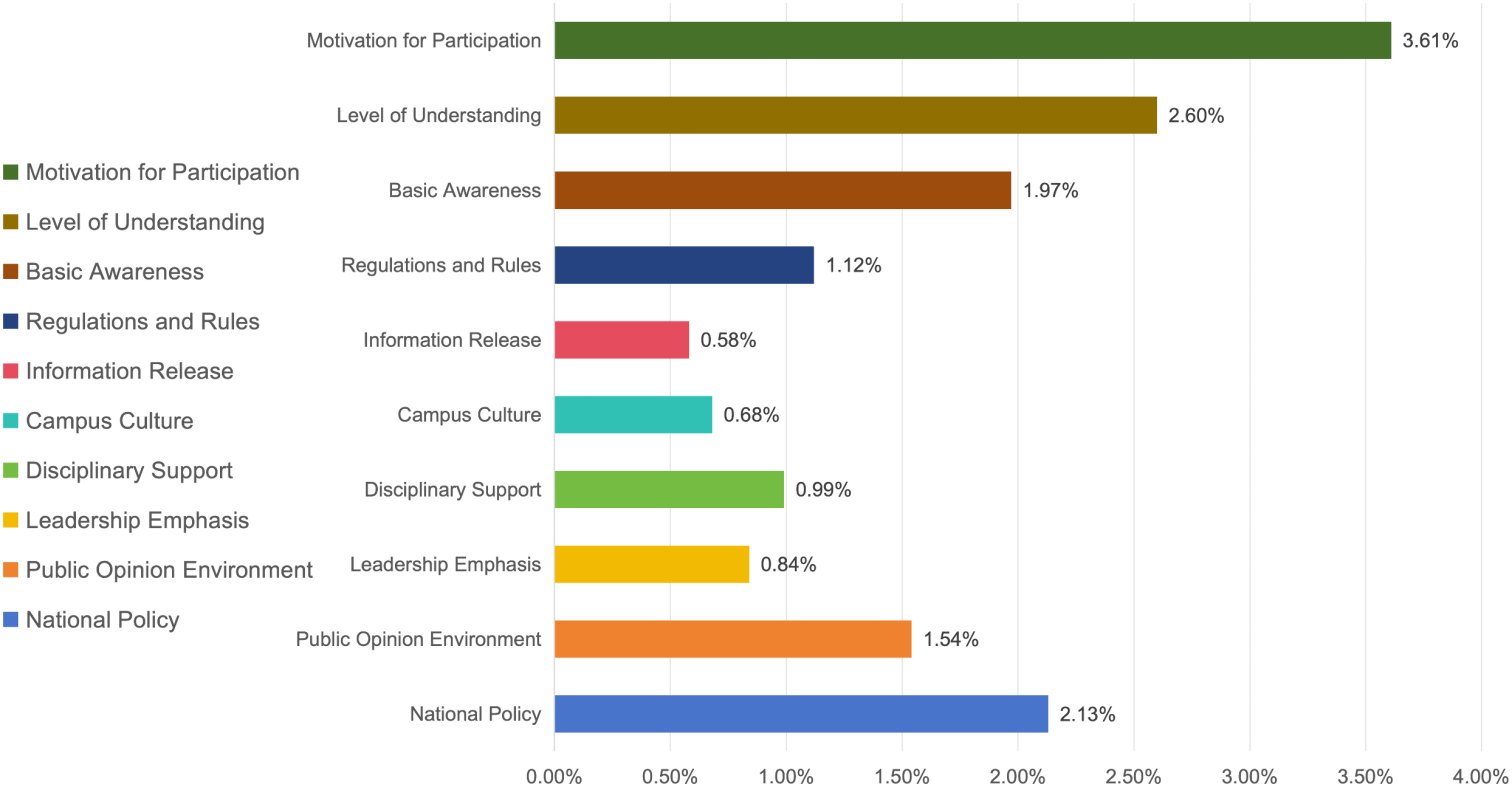
Weights of Tertiary Indicators under the Background of Social Practice.

The background of social practice, serving as the foundational prerequisite for the conduct of practical activities, is assigned the lowest weight among the four primary evaluation indicators. This is largely due to the widespread implementation of social practice, which has gradually been integrated into the educational plan. With the initial establishment of the national policy framework, there is coordination among the state, universities, and society regarding the need to conduct social practice. All parties recognize the importance of practice-oriented education, and there is little difference in their joint support for the implementation of social practice, with the impact becoming more stable. Correspondingly, among the three secondary indicators associated with the background of social practice (Fig 4), student awareness (8.18%) has the highest weight; this weight is significantly higher than that for school environment (4.21%) and social environment (3.67%), making it the key factor in the background of social practice. This indicates that the improvement and effectiveness of social practice cannot be separated from a full understanding of the practice activities performed by the main participants. Universities should help students to recognize the improvements in ideological quality and comprehensive abilities that social practice can bring them before the activities begin. Among the tertiary indicators associated with student awareness (Fig 5), the motivation of students to participate in social practice activities (3.61%), their understanding of the norms and requirements of social practice (2.6%), and their recognition of the importance and necessity of social practice (1.97%) are all important elements affecting student awareness. In addition, the support of national policies for social practice, the guidance of public opinion regarding practice activities, and the regulations and reward and punishment mechanisms implemented by universities regarding social practice are also important prerequisites for the orderly conduct of social practice.

### Discussion on the input indicators and weights of social practice

**Fig 6:**
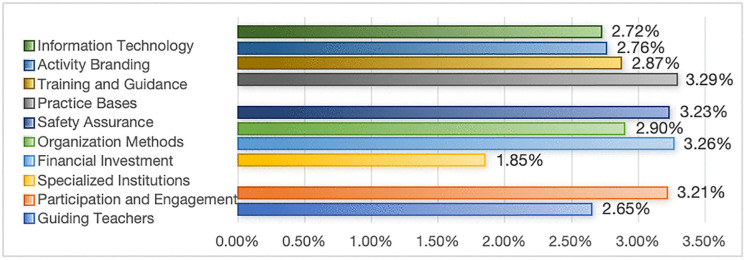
Weights of Tertiary Indicators under the Input of Social Practice.

Input is a crucial component required to conduct practical activities, and serves as the second-highest weighted primary indicator in the evaluation system. During interviews, experts commonly agree that in China’s current phase, efficient input into social practice is essential for achieving more significant outcomes. Only with sufficient faculty, organizational, financial, and training support can social practice be conducted continuously and efficiently. Among these, organizational support (11.24%) and resource support (11.64%) are deemed more important than faculty support (5.86%) (Fig 4). While the involvement of teachers and their organizational guidance is an indispensable element, when building a comprehensive practice-oriented education framework, the efforts of teachers cannot solely be relied upon. Instead, all members must collaborate, in all aspects, throughout the entire process. This requires organizational support to integrate various forces organically and create a synergy, as well as the matching of resources to provide robust conditions for high-quality social practice activities for college students. Among the components of organizational and resource support (Fig 6), financial investment (3.26%), safety assurance (3.23%), and practice bases (3.29%) are relatively highly weighted. In practice, ensuring that the expenses required for students to participate in social practice activities are afforded, providing accidental insurance for teachers and students, conducting safety education and safety plans, and establishing diverse social practice bases that operate regularly are vital for the safe and orderly conduct of social practice activities.

### Discussion on the implementation indicators and weights of social practice

**Fig 7:**
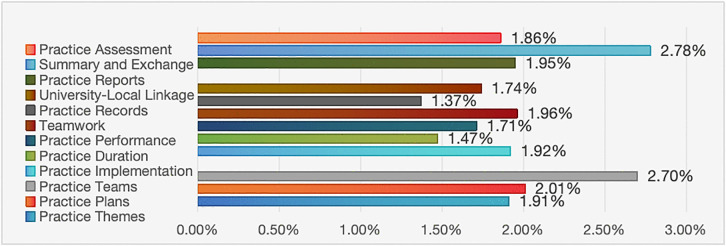
Weights of Tertiary Indicators under the Implementation of Social Practice.

The implementation of social practice is a pivotal component of practical activities, accounting for nearly 25% of the evaluation system. With regard to standardizing social practice activities, practice-oriented education must keep pace with the times, reflect the characteristics of youth, and foster innovation in practice. However, many universities encounter numerous challenges when conducting social practice, such as the uniformity of activity formats, the homogeneity of themes, and the difficulties associated with ensuring that activities endure. The implementation of social practice encompasses three secondary indicators and twelve tertiary indicators. Among the secondary indicators (Fig 4), the weight of the practice process (10.17%) was significantly higher than that of practice design (6.62%) and practice presentation (6.59%). The practice process is not only a crucial method used to implement practice-oriented education, but also one of the most direct and essential means of enhancing educational outcomes. During the practice process, students can integrate their majors and interests to gain insights into the nation’s needs across various domains, such as the economy, society, culture, technology, and ecology. They enhance their knowledge and skills while also stimulating their motivation to learn. Among the tertiary indicators (Fig 7), the practice team (2.7%) pertains to the scientific feasibility of the practice plan and the ability to execute the practice as planned. Summary and exchange (2.78%) are related to students’ capacity to promptly summarize their activities and to internalize and externalize their practical learning through discussions and other methods. Hence, these two indicators are relatively significant, whereas the differences among the other indicators are not substantial and are relatively balanced.

### Discussion on the outcome indicators and weights of social practice

**Fig 8:**
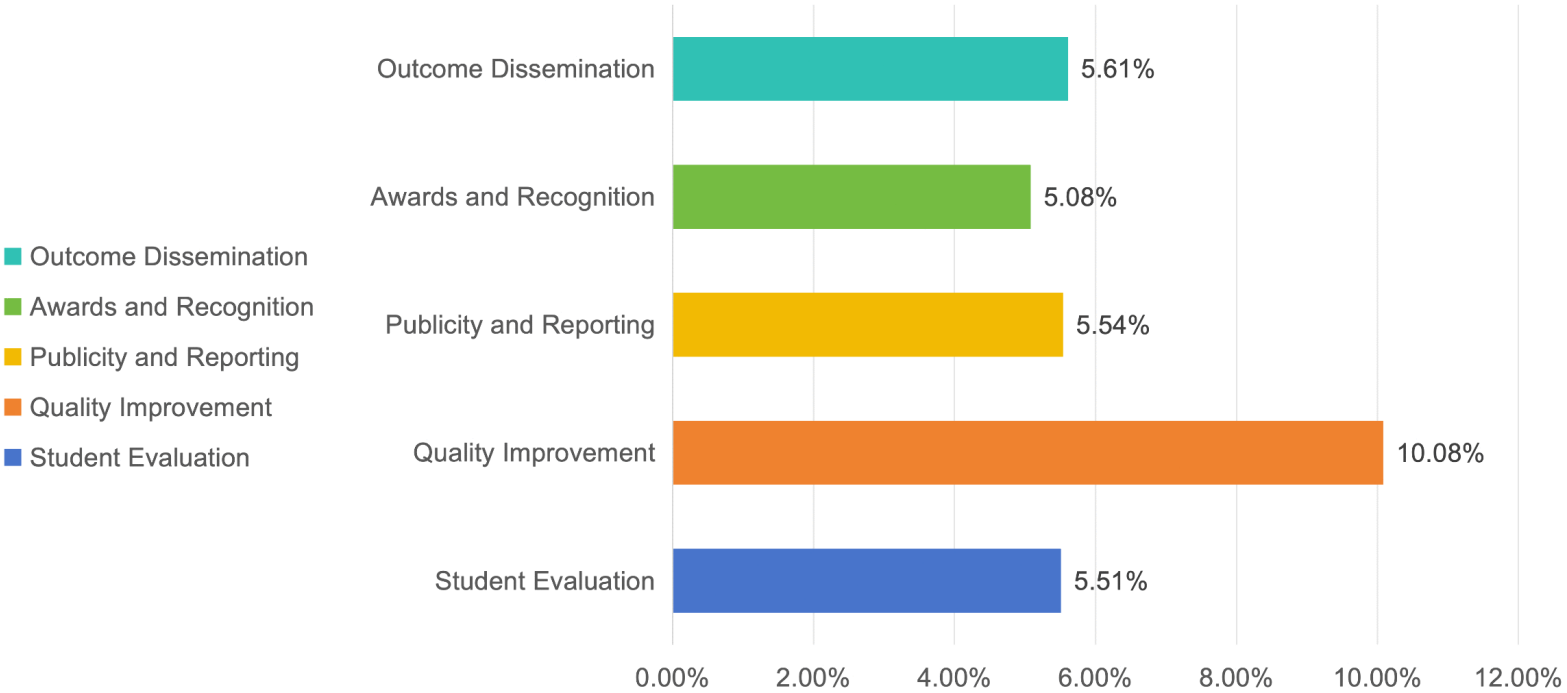
Weights of Tertiary Indicators under the Outcomes of Social Practice.

The outcomes of social practice serve as a crucial foundation for the comprehensive evaluation of practical activities and are assigned the highest weight among the primary evaluation indicators. In alignment with the dual objectives of reinforcing the goal-oriented guidance of practice-based education and overseeing the performance outcomes, a more comprehensive system, deeper meaning, and more substantial results must be obtained for social practice, ensuring that the outcomes of practice become the most significant dimension of observation. Within this, the weight assignments for direct effects (15.59%) and practice impact (16.23%) are nearly equivalent (Fig 4). These two indicators represent distinct facets of the positive influence that social practice exerts on students. Direct effects are gauged from the student self-evaluation perspective, referring to the improvements and satisfaction students experience with regard to their ideological quality, professional skills, and social participation as a result of the practice activities. Practice impact is assessed from the university and societal evaluation perspectives, encompassing media attention and the publicization of practice activities via newspapers, radio, television, and the internet; this also includes the award status of practice outcomes and their adoption by the government, enterprises, and social service departments. Notably, quality improvement (10.08%) holds the highest weight among the 37 tertiary indicators (Fig 8). This aligns with the guiding principle of social practice, which is to guide students towards “receiving education, developing abilities, and making contributions.” Practice serves as a means of education, and education is the goal of practice. Social practice has evolved into an effective approach to enhancing educational practice in Chinese universities. Irrespective of the innovative forms of practice activities, the primary purpose and ultimate goal of improving the effectiveness of practice activities in the new era are guiding students to enhance their quality. By promoting the high-quality development of practice-oriented education, the improvement in student quality is both the starting point and a critical endpoint.

## Conclusions

In this study, the CIPP evaluation model is introduced to assess the implementation of social practice in Chinese universities in a multi-dimensional manner. It constructs a comprehensive evaluation index system for social practice and assigns weights to the indicators using the AHP. This evaluation system is of great theoretical and practical significance for comprehensively evaluating the implementation of social practice activities, improving the organization of activities, and enhancing the effectiveness of practice-oriented education.

Firstly, the research results provide a reference for the comprehensive evaluation of the implementation of social practice in Chinese universities. The selection of indicators in the evaluation system refers to official policy texts and the representative literature, using the CIPP model as a theoretical reference and qualitative interview results as the basis. It covers the entire process of social practice activities. The constructed index system not only focuses on background indicators such as social environment, school environment, and student awareness, but also on input indicators such as faculty support, organizational support, and resource support. It also pays attention to process indicators such as practice design, practice process, and practice presentation, as well as outcome indicators such as the effectiveness of activities and their practice impact. It focuses on both formative and summative evaluations, providing a comprehensive and systematic reflection of the basic situation of social practice in Chinese universities. All indicators have passed the tests of concentration, dispersion, and coordination, showing good applicability and rationality. Chinese universities can consider using this evaluation system to comprehensively evaluate the implementation of social practice to improve the comprehensiveness and accuracy of assessments.

Secondly, the structure and weighting of the indicator system provide a reference for enhancing the performance of practice-oriented education in universities. This evaluation system consists of four primary indicators, eleven secondary indicators, and thirty-seven tertiary indicators. Regarding the weights of the primary indicators, this evaluation focuses on the outcomes and inputs of social practice. The relevant inputs guarantee the provision of practice-oriented education. Regarding the secondary and tertiary indicators, the indicators related to practice-oriented education are divided into internal and external factors. National policies, public opinion environment, regulations, financial investment, and practice bases are external factors that do not vary among individual students and can be quickly improved and enhanced through management measures. Student motivation and personal evaluation are internal factors that are influenced by the preferences of individual students, making external intervention relatively difficult. When conducting practice-oriented education in universities, both external and internal factors should be taken into account, with external factors ultimately contributing to the improvement in students’ comprehensive education through internal factors.

In summary, this study constructs a comprehensive evaluation index system for social practice by conducting a comprehensive and systematic analysis of the multi-dimensional and full-process implementation of social practice in Chinese universities. Based on the results of this study, more in-depth empirical research could be conducted on the implementation of social practice in Chinese universities. This could both verify the conclusions of this study and correct any inappropriate indicator extraction or weighting biases caused by subjective errors or sample limitations in the text analysis, qualitative interviews, and statistical analysis. This will provide references for further enhancing the effectiveness of social practice and strengthening its educational function. In future research, we will focus on promoting the pilot application of this index system. We plan to select several representative universities as cases, use this evaluation system to conduct a systematic assessment of social practice work, and empirically test its practical effectiveness and applicability in identifying problems, guiding improvements, and enhancing project quality.

## Supporting information

S1 Table Lists of policy documents and lists of core articles. (DOCX)
