# Supplementary material for: Research on the comprehensive evaluation index system of social practice in Chinese universities based on the CIPP model
Source: PLoS One. 2026 Apr 24;21(4):e0346058. doi: 10.1371/journal.pone.0346058 (PMC13108873; doi:10.1371/journal.pone.0346058)
Supplement: S1 Table — (DOCX) [file pone.0346058.s001.docx]

**S1 Table. Lists of policy documents and lists of core articles**

**Table 1: Lists of policy documents**

| **No** | **Sources** | **Note** |
| --- | --- | --- |
| **1** | Opinions of the Central Committee of the Communist Youth League and the All-China Students' Federation on Enhancing the Effectiveness of Social Practice Activities for College Students in the New Era and Deepening the Work of Practice-Oriented Education by the Communist Youth League | Published in 2023 |
| **2** | Opinions of the Publicity Department of the CPC Central Committee, the Ministry of Education, and the Central Committee of the Communist Youth League on Extensively, Deeply, and Sustainably Carrying Out Social Practice Activities for College Students | Published in 2020 |
| **3** | Opinions of the Central Committee of the Communist Youth League and the Ministry of Education on Jointly Building the “Grand Ideological and Political” System in Universities and Promoting the High-Quality Development of the Communist Youth League Work in Universities | Published in 2024 |
| **4** | Opinions of the Central Committee of the Communist Youth League and the Party Leadership Group of the Ministry of Education on Innovating and Reforming the Work of the Communist Youth League in Universities to Effectively Enhance the Effectiveness of Ideological and Political Leadership | Published in 2022 |
| **5** | Implementation Outline for the Quality Improvement Project of Ideological and Political Work in Universities | Published in 2017 |
| **6** | Several Opinions of the Ministry of Education and Other Departments on Further Strengthening the Work of Practice-Oriented Education in Universities | Published in 2012 |

**Table 2: Lists of core articles**

| **No** | **Sources** | **Author(s)** |
| --- | --- | --- |
| **1** | The Construction of Evaluation Standard System for College Students’ Social Practice Effectiveness of“Serving the Country People in Three Aspects” | Xuan Fang(2023) |
| **2** | Research on social practice and education in colleges and universities in the new era | Lei Zheng (2023) |
| **3** | On the Model of College Students' Social Practice Education from the Perspective of Curriculum-based Ideological and Political Education | Zhijie Dong(2023) |
| **4** | Analysis of the Effectiveness of Social Practice Activities for University Students and Design of Its Evaluation System | Lu Wang(2023) |
| **5** | The Evaluation and Promotion Strategy of Non-economic Benefits of College Students’Social Practice | Yuping Cui(2022) |
| **6** | Research on the quality improvement of college students’ social practice education | Juan Chen (2022) |
| **7** | Research on the construction of practical education system in colleges and universities in the new era | Lixia Ma (2021) |
| **8** | Research on the Evaluation Index System of the Effectiveness of “Three Rural Programs” Social Practice for College Students Based on the CIPP Evaluation Theory | Baoling Li(2021) |
| **9** | Construction and empirical study on effectiveness evaluation system of social practice education in higher vocational colleges | Chen Zhong(2020) |
| **10** | Research on the Collaborative Education Mechanism of Social Practice in Universities in the New Era | Xiangnian Kong(2019) |
| **11** | An analysis of the educational effectiveness evaluation system of college students’ social practice based on CIPP model | Botong Liang (2018) |
| **12** | An analysis of the educational effectiveness evaluation system of college students’ social practice based on CIPP model | Jinhui Zhang, Botong Liang (2017) |
| **13** | Analysis of the Effectiveness of Social Practice-Oriented Education in Universities | Zhengquan Fang(2017) |
| **14** | Research on the Evaluation Index System of College Students' Social Practice Based on Student Satisfaction | Shujian Xiao(2015) |
| **15** | Analysis of the Effectiveness Evaluation of Social Practice for College Students in the New Era | Hao Ding, Tingting Wang(2014) |
| **16** | Construction of the Social Practice Evaluation System for College Students in Local Polytechnic Universities | Yuguang Zhang, Fengchun Lin, Changhong Guo(2014) |
| **17** | Construction of the Evaluation System for College Students' Social Practice | Jingjie Xu(2014) |
| **18** | Construction of an Evaluation Index System for the Effectiveness of Social Practice Education for College Students | Xihua Liu(2012) |
| **19** | Research on the Evaluation Index System and Evaluation Methods for College Students' Social Practice | Lili Chen(2010) |
| **20** | Reflections on Strengthening Social Practice Activities for College Students | Jirui Yang(2010) |
| **21** | Exploring the Construction of an Evaluation System for College Students' Social Practice | Ping Huang(2008) |
